# Neutrons reveal the dynamics of leaf thylakoids in living plants

**DOI:** 10.1038/s41598-025-22747-z

**Published:** 2025-11-05

**Authors:** Laura-Roxana Stingaciu, Hugh O’Neill, Chung-Hao Liu, Barbara R. Evans, Gergely Nagy

**Affiliations:** 1 Neutron Scattering Division, Oak Ridge National Laboratory, Oak Ridge, TN 37831 USA; 2 Chemical Sciences Division, Oak Ridge National Laboratory, Oak Ridge, TN 37831 USA

**Keywords:** Biological techniques, Biophysics, Plant sciences, Physics, Applied physics, Biological physics

## Abstract

The study is the first known exploration of photosynthetic membranes dynamics in living plants by high resolution quasielastic neutron scattering spectroscopy. We investigated the mobility and flexibility of thylakoid membranes in common duckweed (*Landoltia punctata*) and identified dynamics across various length scales corresponding to individual membranes and membranes stack. We employed classical models typically used to study lipid bilayers to characterize the undulation modes and rigidity of the membranes and reveal how structural variations influence the observed complex dynamics. Our findings show that the stacks of thylakoids in duckweed behave as rigid systems, exhibiting an effective bending coefficient in the lower range associated with surfactant membranes. In contrast, the single thylakoid leaflets display greater apparent flexibility and are well situated within the bi-continuous surfactant phase dynamics. While our observations enhance the understanding of the intricate architecture and mobility of photosynthetic cellular machinery, they also highlight the limitations of applying ideal lipid membranes models to describe complex biological systems. This work opens more questions and the need for further investigations across extended length and time scales, as well as the importance of rigorous sample preparation and experimental control.

**Supplementary Information:**

The online version contains supplementary material available at 10.1038/s41598-025-22747-z.

## Introduction

The light reaction of photosynthesis occurs within the intricate membranous structures known as thylakoids. It has been shown previously in the unicellular cyanobacteria, where the photosynthetic membranes form concentric layers that follow the shape of the cell envelope, that thylakoids are highly flexible structures, and their dynamics is tightly connected with the electron transfer between photosynthetic reaction centers and the associated electrochemical proton gradient across the membrane^1,2^. However, in higher plants the thylakoid membranes are organized in a different fashion. Among other components each leaf cell contains several chloroplasts in which thylakoid membranes are stacked in closed appressed structures ensuring a large area-to-volume ratio and a high stability of the photosynthetic ultrastructure, with remarkable adaptability to environmental conditions^3,4^. To date, detailed information about thylakoids macro-structure in their native environment in leaves is obtained using non-invasive Small Angle Neutron Scattering (SANS)^5–8^. These structural studies suggest that thylakoid membranes within chloroplasts can exhibit a great amount of flexibility and very complex dynamics patterns related to the photosynthetic process^6,8–12^. In respect of that view, our aim was to observe and directly characterize in a non-invasive manner the dynamics of plant thylakoids and assess their flexibility as bending motions and thickness fluctuations as previously done for single-cell photosynthetic microorganisms^1,2^ and standard bilayer phospholipid membranes^13–16^. Neutron Spin-Echo (NSE) spectroscopy is the high-resolution spectroscopic technique that has already proved to be very successful in capturing the structure and dynamics of bilayer lipid membranes with no damage inflicted to membranes^1,13,17,18^. A variety of theoretical models also exist to characterize the bending motions and thickness fluctuations of bilayer lipid membranes^19–24^. The main challenge for this study was to find a suitable plant that could fulfill all the following vital requirements: (i) fast growing in confined lab space to supply plentitude of fresh, intact leaves over a relatively short period of time (ii) survival following extended soaking time in D_2_O, which is necessary to enhance the contrast, (iii) has thin leaves that allow fast gas diffusion and D_2_O exchange of the cell cytoplasmic media, and preferably, (iv) has been investigated by SANS and shows Bragg peaks in the spin-echo accessible *q*-range.

Common duckweed (*Landoltia punctata*, formerly classified as *Lemna min*or) is a small aquatic plant that belongs to the Lemnaceae family. It is one of the smallest flowering plants in the world and can be found floating on the surface of still or slow-moving freshwater bodies like ponds and lakes. Duckweed has tiny, oval-shaped leaves called fronds of a few millimeters in size. Due to its rapid growth and its ability to efficiently absorb nitrogen and phosphorus from the water, duckweed is often used in wastewater treatment^25,26^. Duckweed also provides habitat and food for various aquatic organisms. Its ability to cover large water surfaces can also reduce evaporation and inhibit the growth of harmful algae by shading the water. Duckweed plays a fascinating role in aquatic ecosystems and has practical applications in environmental and agricultural contexts with the potential to be used for biofuel production^27^, protein-rich animal feed^28^ and phytoremediation^29^. In contrast to most terrestrial plants, duckweed can tolerate and grow in relatively high concentrations of D_2_O^30^ as shown by previous studies documenting the production of deuterated biomass^31^ for metabolic studies^32^, and for probing localization of lipid biosynthesis^33^.

For this work we thus try to characterize the mobility of the thylakoid membranes encapsuled by chloroplasts in cell fronds of living duckweed plants by neutron spin-echo spectroscopy, as common duckweed fulfils all the afore-mentioned criteria.

## Results and discussion

### Short description of photosynthetic architecture

**Fig. 1:**
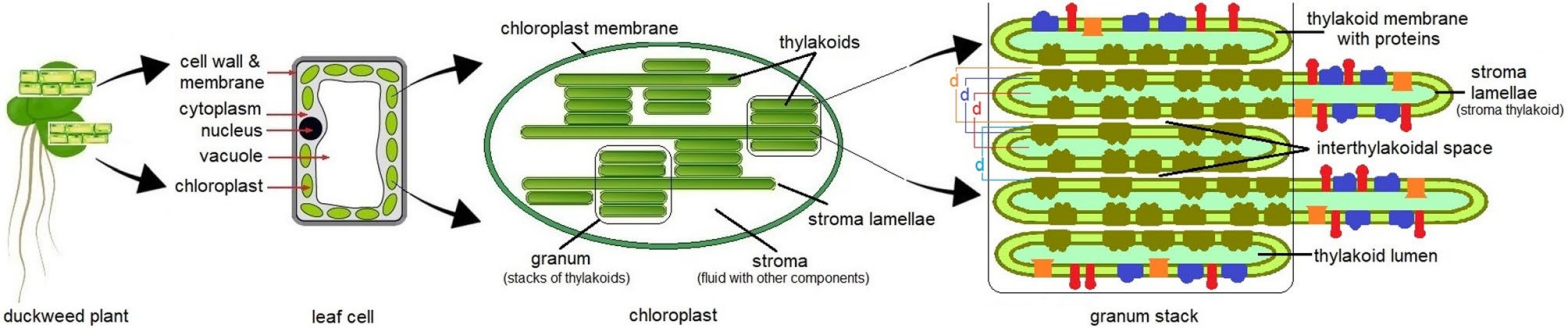
Schematic of duckweed leaf cell structure and chloroplast. Starting from the left side the figure shows the duckweed plant with leaf cells; a single leaf cell enlarged to observe the main cell constituents; the chloroplast enlarged to observe the thylakoidal architecture; and an example of thylakoid membranes stack with several important proteins: photosystem II complex in dark green, photosystem I complex in blue, ATP synthase in red and cytochrome *b*_6_/*f* in orange^35^. *d* is the *repeat distance* (also known as the *film-to-film distance* and *center-to-center distance*)^36,37^. *Note: the dimensions of the different features and the proteins density are not to scale.*

Duckweed has tiny, oval-shaped leaves of a few millimeters in size but very complex in structure. A simplified drawing of the leaf cell and its constituents is presented here in Fig. 1, with additional info in Figure S1 in the Supporting Information. As in all higher plants, leaf cells contain chloroplasts where the photosynthesis takes place. Chloroplasts contain thylakoid membranes arranged in stacks of disks called grana (singular: granum). Grana are connected by stromal thylakoids called lamellae, which join multiple granum stacks together as a labyrinth of chambers forming a single functional compartment. The aqueous space between the thylakoids and the chloroplast membranes is called the stroma. Beside thylakoids, the stroma fluid contains many other components like sugars, chloroplast DNA, ribosomes, various enzymes and starch^34^.

The thylakoids are stacks of connected flattened hollow disks encapsulating the thylakoid lumen and separating it from the rest of the stroma fluid, forming a barrier between two different aqueous phases. The space between two adjacent thylakoid membranes (two disks) is called the interthylakoidal space, measuring approximately ~ 50Å in plant leaves kept in dark, according to literature^38^, see Fig. 1. The interthylakoidal space forms a contiguous aqueous phase with the stroma. The thylakoid membranes have a high density of embedded proteins that form the backbone of the photosynthetic apparatus, like photosystem II complex, photosystem I complex, ATP synthase and cytochromes among others^35^ (colored features in Fig. 1, not at biological scale). Our study was performed in vivo and did not alter the structure of the photosynthetic apparatus. Therefore, in this study “the thylakoid membrane” refers to the entire complex: the lipid membrane with ends enclosed and shaped like a disk plus its anchored photosynthetic proteins.

### Assessment of the photosynthetic structure by SANS

**Fig. 2:**
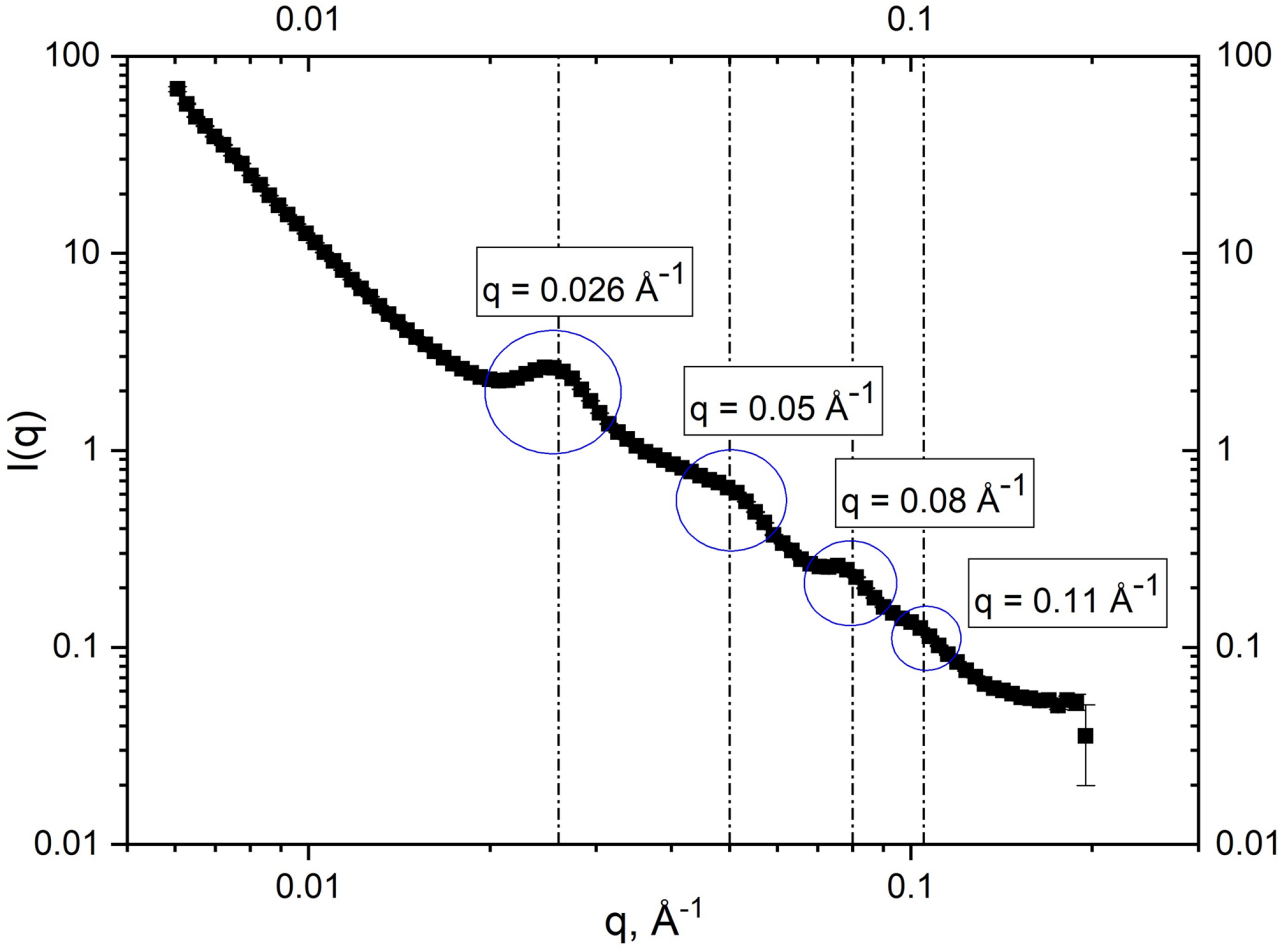
SANS diffraction pattern of thylakoids in intact duckweed leaves. Bragg peaks are observed due to membrane stack periodicity and are visually sampled as *q*-values for the NSE measurement.

Small Angle Neutron Scattering (SANS) was used to characterize the meso-structure and the organization of the thylakoid membranes in duckweed leaves. The result is the scattering curve with several Bragg peaks displayed in Fig. 2.

These characteristic peaks originate from the periodicity of the thylakoid membranes. It has been demonstrated through a series of experiments following isolation protocols of thylakoid membranes from intact leaves^39^, as well as by studies on photosynthetic bacterial cells^40^ and other membrane systems^41,42^, that when a long-range lamellar order exists SANS is the perfect tool to observe the corresponding *repeat distance d* = 2π/*q*, that will give Bragg peaks in the SANS spectrum, with *q* marking the center of the peak. Although the leaf cell structure is very complex (see Fig. 1) and additional complexity comes from the inner chloroplast structure, only strongly ordered features like the thylakoid membranes^42^ will exhibit Bragg peaks in the diffraction pattern. The peak observed at ~ 0.026Å^−1^ relates to the first order Bragg peak of the periodic membrane system, corresponding to *the repeat distance d* ≈ 242Å, and represents also the *film-to-film* distance and average *center-to-center* distance^36,37^ (Fig. 1). Ideally, this Bragg peak would be at the center of our NSE study but unfortunately falls outside the lower *q*-range of the SNS-NSE spectrometer for the 8Å and 11Å incident wavelengths used, where the neutron flux is statistically acceptable. Therefore, as a compromise between detector safety, neutron flux, reliable statistics, and beam-time availability, the next order Bragg peaks in the duckweed diffraction pattern (Fig. 2) were selected for the NSE measurements.

### The dynamics of thylakoids

Neutron spin-echo spectroscopy is a high-resolution scattering technique that measures the time-dependent correlation of atomic configurations. The polarized neutron beam passes through a magnetic field before and after the scattering by the sample, and the velocity of each neutron is encoded in the number of spin precessions (phase angle). The change in neutron energy (velocity) during the scattering by the sample results in an additional phase angle of the corresponding neutron. The total effect on the beam polarization is proportional to the *cos*-Fourier transformation of the spectral function *S* (*Q*, *ω*) called the intermediate scattering function *I* (*q*, *τ*). NSE directly reveals the normalized intermediate scattering function *I* (*q*, *τ*) / *I* (*q*, 0). The accessible correlation time *τ* in the NSE experiment is called Fourier time and is given by:

1 $$\tau \cong \frac{{\gamma \cdot J \cdot m_{n}^{2} \cdot {\lambda ^3}}}{{{h^2}}}$$

with *γ* = the gyromagnetic ratio, *m*_n_ = the neutron mass, *J* = the magnetic field path integral, *λ* = the neutron wavelength and *h* = the Planck constant. The time changes within one experiment run are performed by changing *J* and *λ*. For more detailed explanations of spin-echo spectroscopy technique and its applications we recommend ed. Mezei et al., 2002^43^. Five solid angles determining *q*’s between 0.035Å^−1^ and 0.12 Å^−1^ were set in the spin-echo measurement as *q* minimum values, and *q*’s between 0.04 Å^−1^ and 0.13 Å^−1^ were obtain from the data reduction (*time-of-flight* grouping), that covered most of the Bragg peaks observed in the SANS experiment in Fig. 2. Tuning the *q*’s sampled in NSE to the *q*’s marking the center of the Bragg peaks in SANS is a straightforward way to ensure that most of the signal recorded in spin-echo, hence, the characteristic dynamics observed, will predominately come from the dynamics behavior of the thylakoid membranes ordered within the granum of the chloroplast (see Fig. 1 for the components of the leaf cell and chloroplast). This is our foundational assumption moving forward. Nonetheless, we acknowledge that within the observed *q*-range other structural elements present in the cell (like large proteins unmatched by the deuterated background), could exhibit diffusive motions in the same dynamical range accessed by our measurement, and therefore, contribute to the NSE signal relaxation. Measurements on a much longer time scale, inaccessible by our spectrometer, are usually needed to estimate the contribution of diffusion processes to the overall relaxation.

**Fig. 3:**
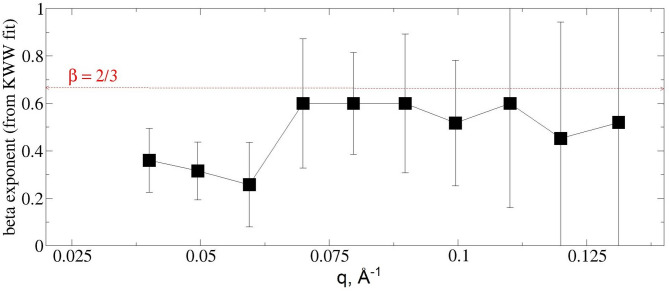
A *q*-dependence of the stretching exponent *β* from KWW fit. The red dotted line is a visual mark for *β* = 2/3 expected for pure undulation.

As the first step in the analysis, the normalized intermediate scattering functions experimentally obtained were fitted by a stretched exponential function with the stretching exponent *β* as a free fitting parameter. The value of exponent *β* can bring insight into specific motions observed by spin-echo, with *β* = 1 indicating translational diffusion, while *β* = 2/3 indicates pure bilayer undulation motion. Figure 3 shows the *q* dependence of the obtained stretching exponent. The values of *β* range between 0.2 and 0.4 in the low q-regime ≤ 0.07 Å^−1^ with the rest of *β* around 0.6 for higher *q* values.

One can also observe the large *β*-errors, providing insights on how the stretched exponential function with the free exponent is not necessarily the correct model to describe the experimental NSE relaxation curves, especially for the data in the highest *q*-regime. Soft upper and lower boundaries (soft limits) were needed in the fit of the stretched exponential function for convergency. The obtained values of *β* exponent, especially in the high *q*-regime, reinforce our foundational assumption that the relaxation we observe by spin-echo is influenced, at least partially, by membranes undulation. Therefore, in the next step of the evaluation the NSE data were fitted by a stretched exponential function with a fixed stretching exponent *β* = 2/3, according to Zilman & Granek^19,20^ model (abbreviated ZG from here on), a traditional model used extensively by the community to characterize lipid bilayers and multilayers membranes dynamics:

2 $$\frac{{{\text{I}}\left( {{\text{q}},{\text{t}}} \right)}}{{{\text{I}}\left( {{\text{q}},0} \right)}}=\exp \left[ { - {{\left( {{\text{\varvec{\Gamma}t}}} \right)}^{2/3}}} \right]$$

were *Γ* is the relaxation rate.

Using this approach for fitting the NSE data any additional diffusion and inter-membrane interactions are neglected. The Zilman & Granek^19,20^ model explains the dependence of the relaxation rate *Γ* with *q*^3^ for systems where the hydrodynamic interactions dominate and the wavelengths are shorter than the characteristic correlation length, in our case *1/q* < < inter-thylakoidal distance of about ~ 50 Å^38^:

3 $$\Gamma _{{ZG}} = 0.025\alpha \sqrt {\frac{{k_{B} T}}{{\tilde{\kappa }}}} \cdot \frac{{k_{B} T}}{{\eta _{{D2O}} }} \cdot q^{3}$$

*Γ*_ZG_ is the decay rate due to pure undulation fluctuations obtained from the linear fit of all relaxation rate *Γ*/*q*^3^ values, with slope = 0 and intercept value *y*_0_ = *Γ*_ZG_/*q*^3^, therefore, a weighted average of relaxation rates; $\:\stackrel{\sim}{\kappa\:}$ is the effective bending modulus, *α* is an empirical angle factor = 1 for NSE, *k*_B_*T* is the thermal energy, and *η*_D2O_ is the viscosity of the D_2_O solvent at *T* = 20 °C.

**Fig. 4:**
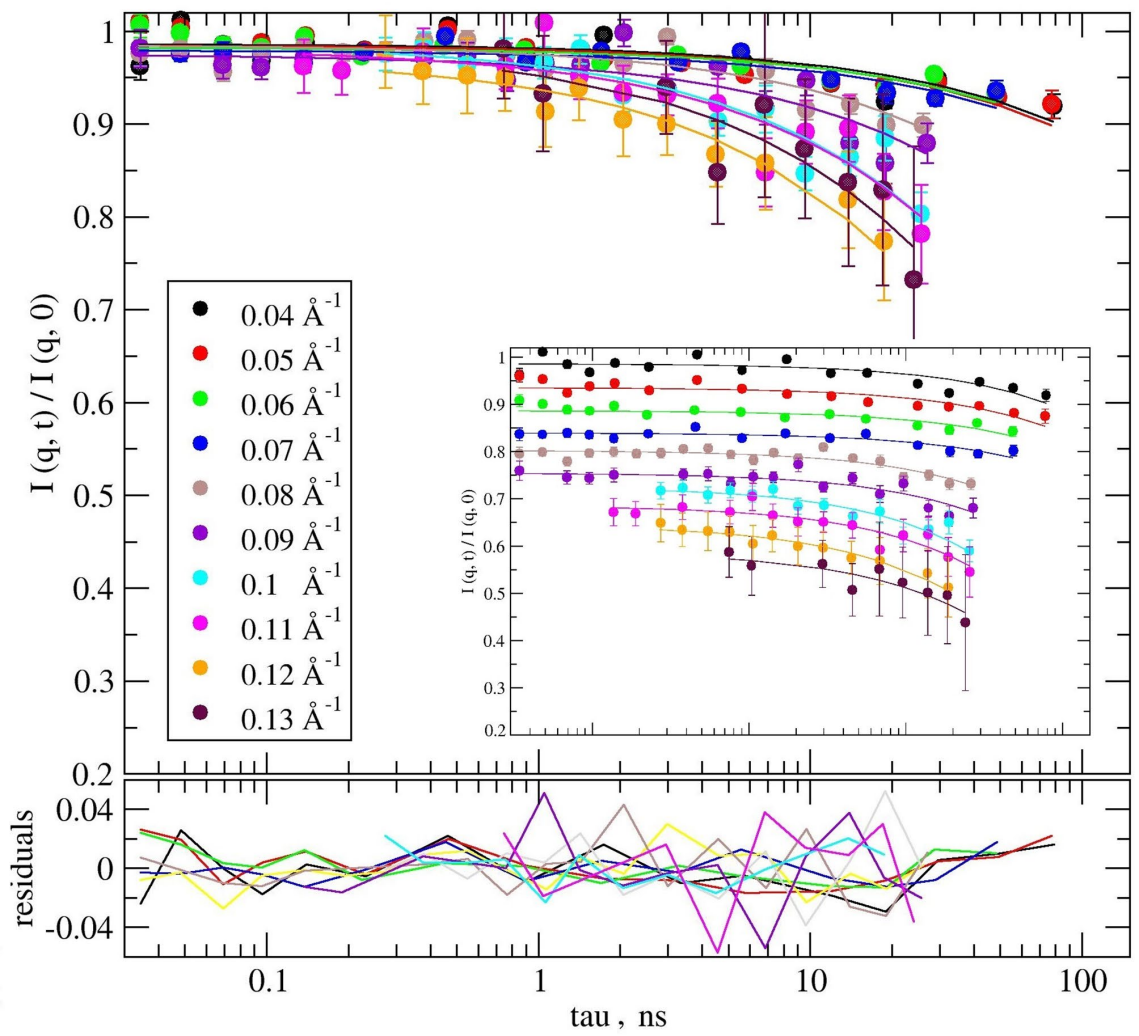
Experimental *I* (*q*, *τ*) / *I* (*q*, 0) of duckweed modeled by ZG. Solid lines in same color represent the stretched exponential function fitting with the stretching exponent of 2/3, according to ZG model. In the inset the scattering functions have been shifted for better visualization. The lower graph is the fit residuals.

**Fig. 5:**
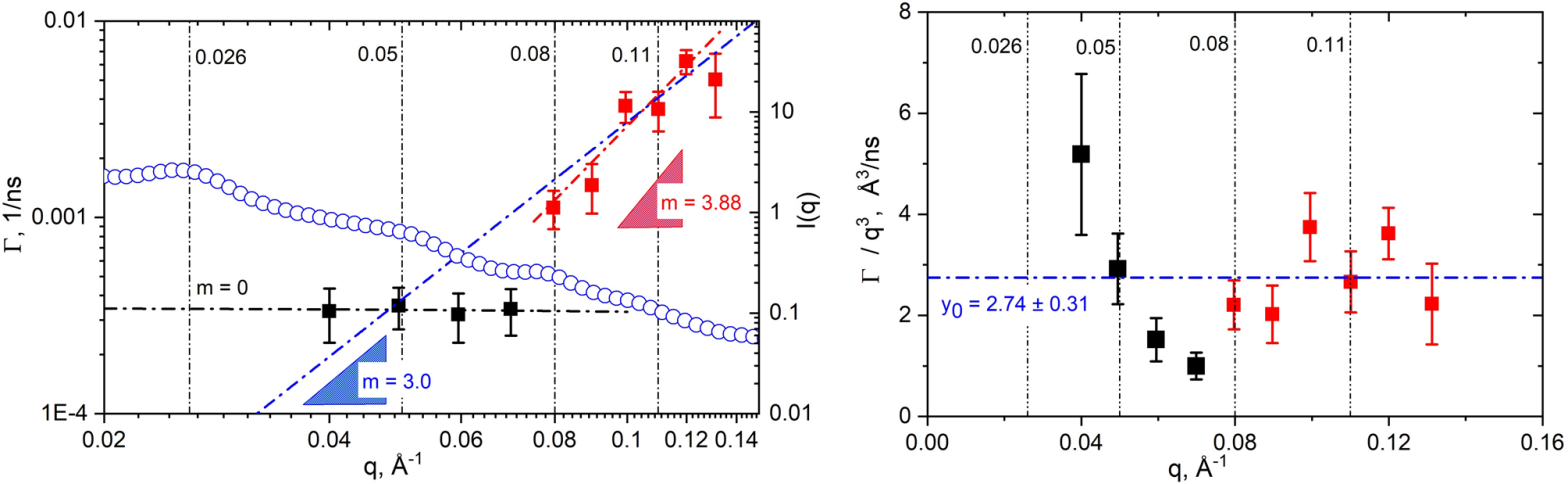
A *q* and *q*^3^ dependence of the decay rate *Γ*. In both panels the vertical dashed lines mark the positions of the corresponding Bragg peaks, from the SANS profile *I*(q) superimposed in blue circles in the left panel (please see Fig. 2 for the full *I*(q) spectra). Linear regression fit was applied to the data on the left-side panel using only the low *q*-range (*q* ≤ 0.07 Å^−1^) *Γ* values (black), and only high *q*-range (*q* > 0.07 Å^−1^) *Γ* values (red). A linear regression with slope m = 3 is added for comparison (blue). In the right-side panel, the horizontal blue line represents the linear fit of relaxation rates *Γ*/*q*^3^ values for the high *q*-range > 0.07 Å^−1^ with slope = 0 and intercept *y*_0_ = *Γ*_ZG_/*q*^3^, used to calculate the apparent bending modulus of the membranes, according to Eq. (3).

The ZG model provides a reasonable description of all the NSE experimental data, with χ^2^ = 0.019 per degree of freedom, see Fig. 4. The corresponding information obtained can be expressed either as relaxation time *τ* or inversely, as relaxation rates *Γ*, using Eq. (2). In the following, we used the spatial dependence of the relaxation rate *Γ* to characterize the dynamics of our system. The *Γ*(*q*) shows different patterns between the low *q*-range (black symbols) and the high *q*-range (red symbols) in Fig. 5, left panel.

In the low *q*-range, there is almost no change in the relaxation rate values as a function of *q*. This happens usually in systems where local dynamics are dominated by interactions that are uniform across distance, leading to a lack of spatial dependence. Saturation effects can happen, and the membrane response is no longer sensitive to changes in *q*, *e*.*g*., all pathways for membranes relaxation were utilized and varying distance no longer influences the relaxation rate. A slope of m = 0 for Γ(*q*) indicates that the primary relaxation is dominated by non-diffusive processes, heavily influenced by structural changes or local rearrangements that are not dependent on distance. This relaxation in confined geometry is relevant when considering the architecture of the thylakoids closely appressed in stacks and enclosed in chloroplast capsules (see Fig. 1) that have restricted space for free undulation and lateral diffusivity. An average relaxation time of more than 2800 ns can be computed in this region, too slow for what the NSE technique can routinely measure today.

In the high *q*-range > 0.07 Å^−1^, the relaxation rate is no longer constant but characterized by a power law with the exponent of *m* = 3.88. A linear regression with the slope *m* = 3 was added for comparison in Fig. 5, left panel. The m = 3 slope is a strong indication of bending undulations observable in that regime and describes well the high *q* data with *R*^2^ = 0.85.

The original ZG model was developed and used extensively to characterize relaxation in ideal lipid membranes, with validation in the high *q*-regime (in our case at 1/*q* < < interthylakoidal distance) where it will appear as a linear dependence of the *Γ*/*q*^3^ of *q*, the typical signature of undulation motions in lipid bilayers. Given the value of the obtained *Γ*(*q*) slope in the range of *m* ≈ 3, we also chose to present our *Γ*/*q*^3^ dependence of *q* to observe better its behavior at high *q*-values, in Fig. 5 right panel. It shows a poor linearity between the relaxation rates *Γ*/*q*^3^ at *q* > 0.07 Å^−1^, with scattered data and large error bars. The deviations observed imply, on one hand, additional relaxation due to superimposed dynamical processes, on top of the simple undulation motions of lamellar lipid membranes. On the other hand, the deviations observed imply also the limitations of the ideal ZG model for characterizing bending fluctuation in complex architectural membrane structures like the thylakoids. The nature of this chaotic relaxation pattern arises from the complexity of the thylakoids and their intricate dynamic behavior = multiple lipid membranes with embedded and free proteins, stacked and appressed within the chloroplast. The photosynthetic membranes closely appressed together in stack have limited space for relaxation; when relaxing they detect the presence of other neighboring membranes in terms of fluctuation dynamics, *e*.*g*. strong structural interactions within the stack. One cannot easily deconvolute the single bilayer membrane undulation from the overall thylakoid-thylakoid dynamics interactions. A multitude of other components besides the single bilayers (large proteins, organelles, etc.,) are also present in the chloroplast and certainly contribute with additional relaxation modes. The value of the *Γ*(*q*) slope of m = 3.88 (close to 4) is a solid indication of changes in the dynamics related to the presence of additional constraints or interactions. Several potential explanations for a relaxation slope closer to m = 4 include: the viscoelastic properties of the membranes, collective dynamical effects among the membrane constituents, caging effects where membranes motion are temporarily hindered, and anomalous diffusion. Nonetheless, classical ZG model can still give a good initial qualitative and quantitative estimation of the bending dynamics.

### Characteristics of bending motions

In typical lipid bilayer membranes that exhibit undulation motions the *q*^3^ dependence of the relaxation rate *Γ* is controlled by the elastic modulus $\tilde {\kappa }$, with higher bending coefficients corresponding to smaller relaxation rates^19,21^. The effective bending coefficient was calculated according to Eq. (3) from the intercept value *y*_0_ = *Γ*_ZG_/*q*^3^ in Fig. 5, right panel (the linear regression with slope = 0 depicted by the blue dashed line, using only the *Γ*/*q*^3^ at high-*q* values, at *q* > 0.07 Å^−1^) and expressed in units of thermal energy and presented in Table 1. The large value of $\tilde {\kappa }$ ≈ 872 *k*_B_*T* indicates a rigid system. Bending rigidity in the range of hundreds of *k*_B_*T* is typical for: highly structured vesicles, liposomes with specific compositions, specialized membranes like those involved in viral enveloping, certain types of organelle membranes, or membranes with high concentrations of macromolecules grafted on the surface that act like fluctuation suppressors^19,21^. All these are expected considering the architecture of the thylakoid stacks in the cells of duckweed leaf. The effective bending coefficient in stacked membranes depends also strongly on the viscosity of the solvent confined between membranes (in the interthylakoidal space), as shown previously in several studies^15,21^. A rescaling of the bulk D_2_O viscosity, *η*_D2O_, to 3·*η*_D2O_ as suggested by these studies brings the effective bending coefficient of the thylakoids stack to $\tilde{\kappa }$ ≈ 97 *k*_B_*T* (see Table 1), toward a reasonable lower range for surfactant membranes, where values within an extensive range of 0.1–2000 *k*_B_*T* have been reported. Rescaling the bulk D_2_O viscosity holds some biological relevance for leaves thylakoids, since the interthylakoidal space is part of the stroma = the fluid where the light-independent reaction process of photosynthesis takes place like the carbon cycle. After the carbon cycle expels glucose, this specialized sugar stays in the stroma until it is needed for uptake, therefore increasing the fluid viscosity together with other stroma components like enzymes and starch.

**Table 1:** Characteristics of bending fluctuations.

Sample name	Γ _ZG_ / q^3^ (Å^3^/ns)	$\tilde{\kappa }$ (k_B_T)	$\tilde{\kappa }$ for 3 · η_D2O_ (k_B_T)
Duckweed	2.74 ± 0.31	871.9 ± 290.6	96.9 ± 32.3

The bending modulus was calculated for *η*_D2O_ = 0.00125 kg m s^− 1^ as D_2_O viscosity at 20 °C, and for 3·*η*_D2O_ as suggested by literature^15,21^.

### Characteristics of local fluctuations

**Fig. 6:**
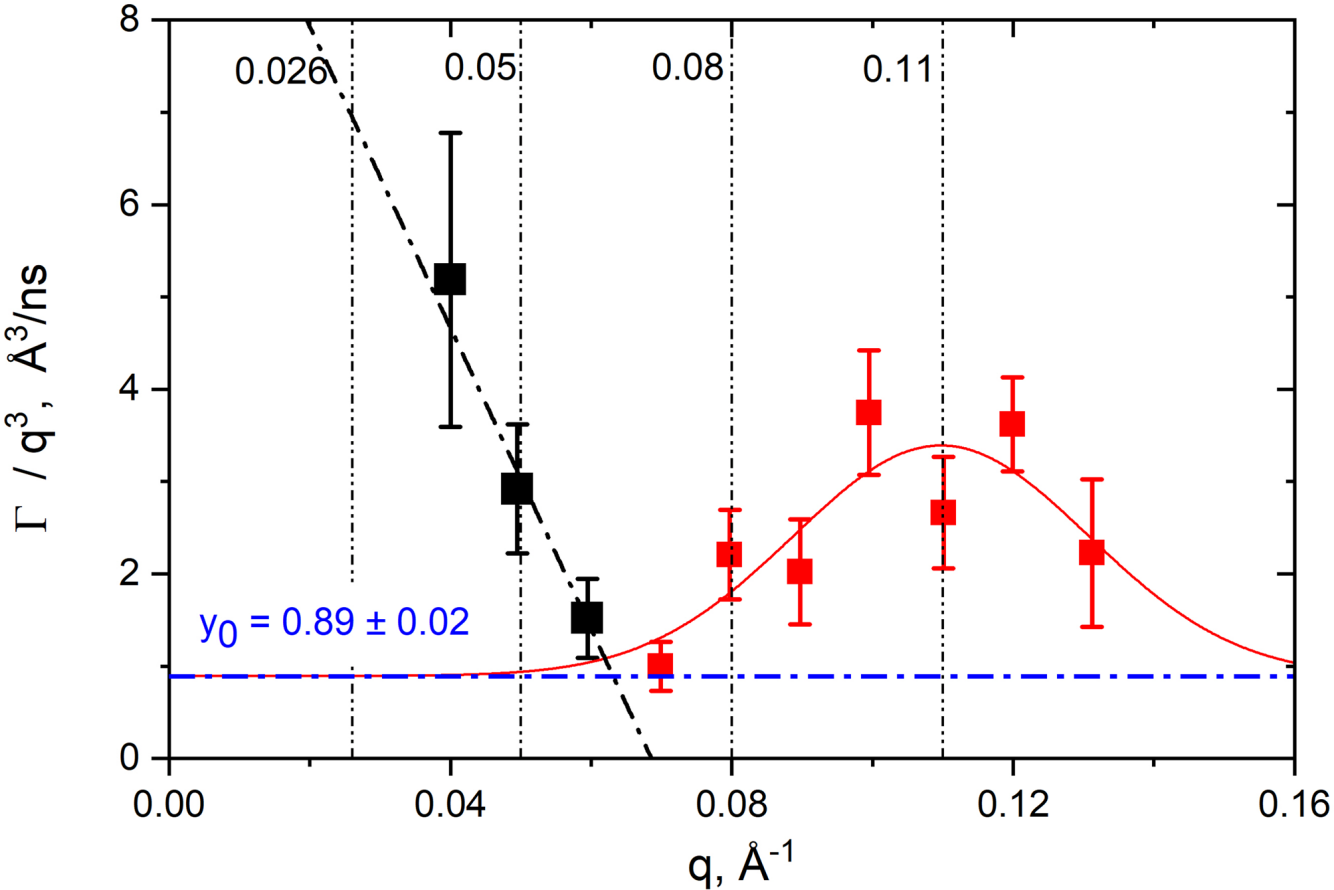
Empirical Lorentz description of excess dynamics attempted on the *Γ*(*q*^3^). The vertical lines show the position of the corresponding Bragg peaks. The horizontal blue line represents the *y*_0_ intercept value of the Lorentzian fit used in the calculation of thickness fluctuations.

The spatial dependence of the decay rate *Γ* shows excess mobility in addition to the underlying *q*^3^-dependent undulation dynamics. To quantify these deviations we continue the analysis of *Γ(q*^3^) with the approach established by Nagao and collaborators^14,22^ where the excess motion is described as local shape and thickness fluctuations with peristaltic and protrusion motions, using a well-known analogy with the shape fluctuations of droplet micro-emulsions^44^:

4 $$\frac{{\text{\varvec{\Gamma}}}}{{{{\text{q}}^3}}}=\frac{{{{\text{\varvec{\Gamma}}}_{{\text{ZG}}}}}}{{{{\text{q}}^3}}}+\frac{{\text{A}}}{{1+{{\left( {{\text{q}} - {{\text{q}}_0}} \right)}^2} \cdot {{\text{\varvec{\upxi}}}^2}}}$$

where: *Γ* is considered the effective relaxation rate due to both bending motions, *Γ*_ZG_, and local thickness fluctuations, *Γ*_TF_, with *A* the damping frequency of the peristaltic mode, *A* = *Γ*_TF_/*q*_0_^3^, *ξ* is the mode amplitude and *q*_0_ defines the membrane thickness at which the excess motions associated with local shape fluctuations are observed^44^. There are two strong deviations identifiable in our *Γ*(*q*^3^): one in the low *q*-range where we sample distances proportional to the *center-to-center* bilayer thickness, and another deviation in the high *q*-range at distances proportional to the single leaflet thickness and interthylakoidal space thickness. In the past, similar excess dynamics have been attributed to swelling of the membrane pair, e.g., thickness fluctuations, bilayer-bilayer interactions, and dynamics due to protrusion and diffusion of proteins at the membrane surface^22,45,46^. For this analysis the relaxation rate *Γ* was separated again into two *q*-regimes: a low *q*-regime < 0.07 Å^−1,^ black data in Fig. 6, and a high *q*-regime ≥ 0.07 Å^−1^, red data in Fig. 6. A linear regression was fitted to the low *q*-regime and a Lorentzian was used for the analysis of the high *q*-data.

The dynamics occurring at length scales corresponding to bilayer periodicity in the low *q*-regime < 0.07 Å^−1^, is best described by a linear regression, consistent with local dynamics dominated by uniform interactions across *q*, leading to a lack of spatial correlation. The faster normalized decay rate *Γ*/*q*^3^ translates into longer relaxation times (*i*.*e*., 2800 ns) and, therefore, slower motions. The slow, constant motions, observable in this *q*-range could be attributed to the progressive swelling and deflating of the thylakoid pair. The swelling of the pair happens within the rigid constraints of the membranes stack. The photosynthetic membranes are living structures, and a certain amount of flexibility is needed to adjust to photosynthesis, respiration, redistribution of proteins and organelles, and other local rearrangements that are *q* independent.

**Table 2:** Characteristics of local thickness fluctuations.

Lorentz fit parameters	Γ/q^3^ intercept (Å^3^/ns)	q_0_ (Å^−1^)	A (Å^3^/ns)	ξ (Å)	A*q_0_^3^ × 10^− 4^ (ns^− 1^)	χ^2^
	0.89 ± 0.02	0.1097	0.13 ± 0.03	0.04 ± 0.01	1.74 ± 0.04	0.42

*A* = damping frequency of the peristaltic mode, *ξ* = the peristaltic mode amplitude, *q*_0_ = the peak maximum position, local length scale of thickness fluctuations. The product *A*·*q*_0_^3^ describes the decay rate of the local fluctuations. *Note that the parameters are calculated for the peak displayed in Fig. 6*using the intercept value y*_*0*_ *= Γ/q*^3^ *= 0.89 of the Lorentzian.*

The dynamics occurring at length scales corresponding to single leaflet thickness in the high *q*-regime ≥ 0.07 Å^−1^ is best described by a Lorentzian with the peak center situated around *q* = 0.1097 Å^−1^, coincidently, the position of the last observable Bragg peak. The calculated parameters from the Lorentz fit are presented in Table 2. The fast decay rate of the local thickness fluctuations *A*·*q*_0_^3^ = 1.74·10^− 4^ ns^− 1^ gives an average relaxation time of more than 5700 ns, double than the average relaxation time of 2800 ns computed for the low *q*-region, and again too slow for the NSE experimental window. The region corresponding to single leaflet thickness is therefore characterized by slow motions with very small amplitude (the peristaltic mode amplitude = 0.04 Å). The decay rate of the local fluctuations *A*·*q*_0_^3^ describes motions within short distances in the range of the single leaflet membrane ~ 50 Å, *i*.*e*, short-range dynamics due to protrusion and diffusion of large proteins on the surface of the single membrane leaflet. This contributes with additional relaxation modes and contaminates the pure undulation of the membranes expected in this *q*-range, leading to a complex relaxation pattern that cannot be easily deconvoluted. The Lorentzian profile indicates that there is a characteristic relaxation time (or frequency) at which the system responds most vigorously. Other processes that can overlap with the undulatory pattern of the membrane and complexify its dynamics as a Lorentzian relationship are critical phenomena like phase transitions, collective excitations, spin relaxation in paramagnetic components, diffusion of gases through the membranes, etc., all of them highlighting the non-trivial dynamics of biological reality than cannot be oversimplified by ideal models and may fail to capture the full scope of biological processes.

### Analogy with lamellar microemulsions

The thickness fluctuation parameters described previously originate in an analysis based on the shape fluctuations of droplet micro-emulsions, used successfully by Nagao^22^ and Milner^44^ among others. Experimental work on lamellar microemulsion of sodium dodecyl sulfate (SDS) of Safinya et al.^47^ showed that for intermembrane distances between ~ 40 Å and ~ 170 Å Helfrich steric interaction is a dominant interaction. The *q-*window of our sample Bragg peaks is 0.026 Å^−1^ ≤ *q* ≤ 0.11 Å^−1^, with the NSE measurements on duckweed accessing *q*’s in the range of 0.04 Å^−1^ − 0.13 Å^−1^ and sits within the exact region of steric repulsions described above. With increasing distance from the last Brag peak marked at high *q* ≈ 0.11 Å^−1^ (*d* ≈ 50 Å) to the first peak marked at low *q* ≈ 0.026 Å^−1^ (*d* ≈ 242 Å) in Fig. 2, the field of view changes from the local single membrane leaflet to the *center-to-center* repeat distance of adjacent bilayer pair within the stack. The thermal fluctuations can induce collisions between neighboring membranes. These collisions give rise to repulsive bilayer-bilayer interactions caused by the reduction of entropy, the Helfrich steric repulsion^48^. Therefore, we made an analogy between the observed excess dynamics in thylakoids and the dynamics related to shape fluctuations in oriented lamellar microemulsions^49,50^. Mihailescu et al.., model calculates the dynamics of a stack of oriented lamellae (the coherent intermediate scattering function arising from oriented lamellar phases) at the length scale of the intermembrane distance^49,51^, as opposed to the classical ZG model applicable only to single bilayers. The model is implemented in the software Jscatte*r*^52,53^ and yields information about the apparent bending elastic modulus, ${\text{\varvec{\upkappa}}}$, and the dissipation related to the viscosity of the solvent *ρ*, according to Eq. 26 in Mihailescu et al.^49^:

5 $$S\left( {q,t} \right) \propto \mathop \smallint \limits_{0}^{{{\rho _{max}}}} d\rho \rho {J_0}({q_ \bot }\rho ){\text{exp}}\left\{ { - \frac{{{k_B}T}}{{2\pi {\text{\varvec{\upkappa}}}}}{q^2}{\mu ^2}\mathop \smallint \limits_{{{k_{min}}}}^{{{k_{max}}}} \frac{{dk}}{{{k^3}}}\left[ {1 - {J_0}\left( {k\rho } \right){e^{ - {\omega ^\infty }\left( k \right)t}}} \right]} \right\}$$

**Fig. 7:**
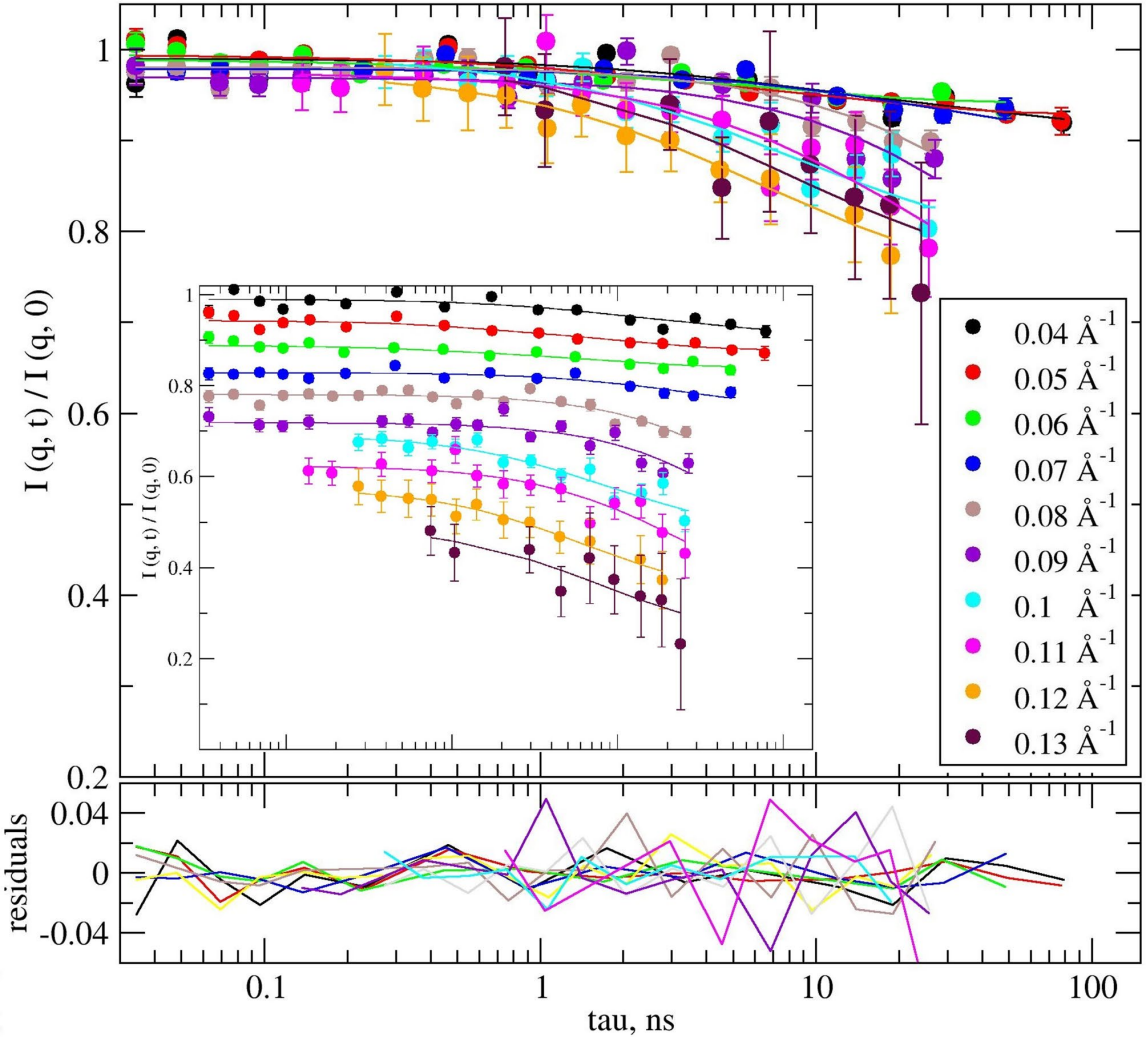
Experimental *I* (*q*, *τ*) / *I* (*q*, 0) of duckweed modeled by oriented lamellar phases. Solid lines represent the fitting by the dynamics of oriented lamellar phases model according to Mihailescu et al., 2002^49^, see Eq. (5). In the inset the scattering functions have been shifted for better visualization. The lower graph shows the fit residuals.

In the limit of large bending rigidities (${\text{\varvec{\upkappa}}} \gg$ *k*_B_*T*), Eq. (5) from above reduces to a simple stretched exponential function, e.g., the ZG model or membrane Zimm dynamics. In our case the model was applied to determine the apparent bending modulus in thermal energy units, the membrane thickness, and the solvent viscosity, using the *film-to-film* distance *d* ≈ 242Å (from first SANS peak @ *q* ~ 0.026Å^−1^) representing the periodicity of the structure or repeat distance as an input parameter. The fit is shown in Fig. 7. The best fit was obtained assuming *q*-dependence of the bending modulus and solvent viscosity, with χ^2^ = 0.017 per degree of freedom (117 dof, with 31 parameters fitted simultaneously for the ten experimental scattering functions, *i.e. q*’s).

**Fig. 8:**
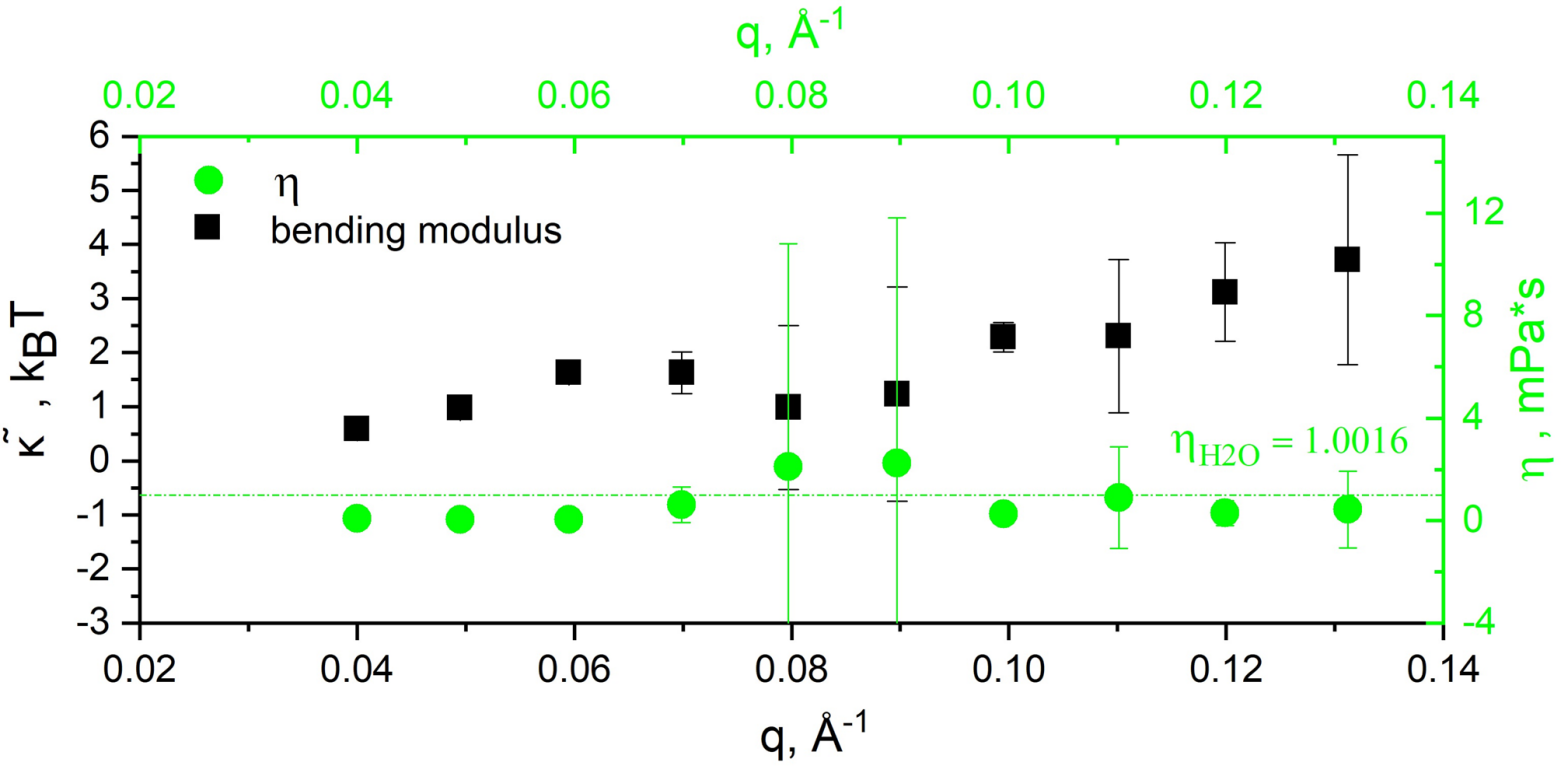
The single membrane apparent bending modulus (black) with superimposed solvent viscosity (green). Values were calculated using the dynamics of oriented lamellar phases model for T = 20 °C and a *film-to-film* distance ≈ 242Å corresponding to the first SANS Bragg peak. The dashed green line represents H_2_O dynamic viscosity ~ 1 mPa*s.

The apparent bending modulus calculated as a function of *q* varies between 0.59*k*_B_*T* and 3.72*k*_B_*T*, see Fig. 8. In comparison to the ZG model that provides only an effective bending modulus, $\tilde{\kappa }$, in the order of hundreds of *k*_B_*T* (Table 1) as a more generalized property based on theoretical considerations, the apparent bending modulus calculated here describes the observed behavior of the thylakoids in the specific conditions of the NSE experiment, with values well in the range of bi-continuous surfactant phases^51^ of ${\text{\varvec{\upkappa}}}$ ≈ 1.3–1.5 *k*_B_*T*. The single bilayer thickness calculated is 65 Å ± 24 Å, strongly corelated with the solvent viscosity. In the analysis, bulk apparent viscosity was assumed and fitted for each *q* to account for local viscosity fluctuations, e.g., local membranes heterogeneity. These variations can be seen in Fig. 8, the viscosity plot superimposed in green symbols. An average value of 0.75 mPa*s (with a large standard deviation of 0.75) can be calculated from the viscosity values, falling well within the range observed for the n-alkanes and chloroalkanes, as well as aqueous solution with high solutes concentration^54^. We theorize that the sensitivity of the calculated parameters (bending modulus and bilayer thickness) to bulk viscosity represents a qualitative description of the local viscosity fluctuations due to thylakoid architectural heterogeneity (the presence of proteins, sugar particles and starches within the stroma fluid and in the interthylakoidal space, variations in lipids concentration, increased local friction with the solvent, etc.,). Fluctuations in these viscoelastic properties of the membranes alter the local dynamics and the apparent rigidity observed by NSE.

### A short summary of the findings

Based on the single study presented here, any definitive conclusions about the behavior of chloroplast photosynthetic membranes are premature. Cautionary, we conclude that insights into the dynamics of photosynthetic membranes are possible using neutron spin-echo spectroscopy. The models used for data interpretation were developed for bilayer lipid membranes and surfactant systems, but they can still provide initial qualitative and qualitative information about the flexibility of thylakoids and their environment.

The heterogeneous behavior of the membranes relaxation rate suggests a lack of correlation due to processes affecting relaxation across distances, saturation of relaxation mechanisms, effects related to the presence of additional constraints or interactions, as well as limitations in the data and spectrometer resolution. The thylakoid undulation is contaminated by viscoelastic effects, collective dynamics, restricted movement due to crowding, short-range dynamics due to protrusion and diffusion of large proteins on the surface of the single membrane leaflet. All these factors suggest a more complex interplay of molecular mechanisms at work, compared to the simpler, ideal case of bilayer phospholipid membranes extruded in controlled lab environment.

We observed rigid thylakoids with effective bending coefficient in the range of highly structured vesicles, liposomes, or specialized membranes with high concentrations of macromolecules grafted on the surface, expected considering the architecture of the thylakoid stack. The apparent bending moduli calculated at the length scale of the intermembrane distance sits well in the range of bi-continuous surfactant phases, with sensitivity to local heterogeneity.

Future planned experiments at extended *q*-range with the characteristic dynamical trends, combined with a more thorough analysis of the Bragg peaks under variable illumination conditions are planned, and we hope to expand further our understanding of the dynamics of the extremely complex photosynthetic machinery in leaves.

## Methods

### Sample preparation and setup

**Fig. 9:**
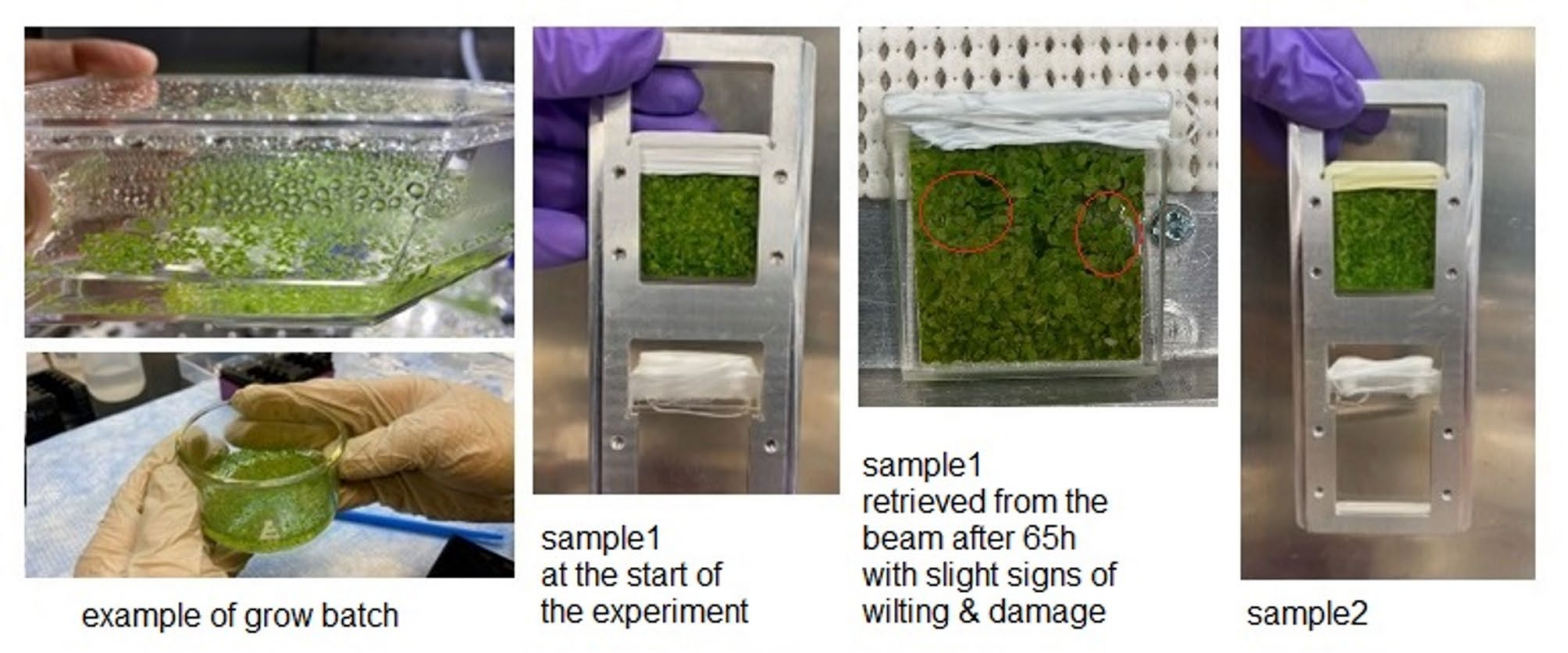
Duckweed sample preparation. Left-side panels show representative batches during growing and harvesting phase; Middle and right-side panels show the two NSE samples collected in quartz cells and mounted on the NSE sample holder frame together with the D_2_O solvent sample. Note that the NSE samples were interchanged in the middle of the NSE experiment (after ~ 65 h) to ensure fresh living plants for the entire length of the measurement. Two identical samples were prepared consecutively and swapped during NSE measurements to make sure the plants are alive. The samples were visually inspected at regular intervals (daily) for signs of wilting and leaves breakage. When those slightly occurred, after approximatively 65 h, the first sample was retrieved and replaced with the second sample to ensure fresh and potent plants, see Fig. 9. No major wilting signs or sample decomposition was observed for the duration of the experiment (130 h). Please see the Supporting Information, Figure S4, for additional bench-top testing of the viability of the samples after extended soaking time in D_2_O.

Duckweed (Landoltia punctata 6-DW138, formerly classified as Lemna minor) was purchased from the Rutgers Duckweed Stock Cooperative (Rutgers State University New Jersey, New Brunswick, New Jersey, USA). The plants were grown and allowed to multiply in H_2_O-based medium (3.2 g/L Schenk and Hildebrandt Basal Salt Mixture at pH4.2, Millipore Sigma, USA) at 24 °C and 100 µE light intensity with a 12 h/12 h day/night cycle. Before the NSE experiment, the plants were transferred from the grow media to D_2_O, equilibrated overnight for ~ 12 h, and the batches were combined in a final sample batch. The duckweeds were gently harvested by spatula and placed in NSE quartz cells of 4 mm thickness, as shown in Fig. 9. In the final step, pure D_2_O was added as solvent directly in the NSE cell, and a couple of hours of equilibration were allowed for extra D_2_O exchange, to further improve the contrast. By this process we believe that all the exchangeable Hydrogen atoms were replaced by Deuterium atoms.

### SANS experiment and data reduction

The SANS results were collected at the Extended Q-Range Small-Angle Neutron Scattering Diffractometer (EQ-SANS) at the Spallation Neutron Source, Oak Ridge National Laboratory^55^. The instrument configuration was in 60 Hz operation mode, using 4 m sample-to-detector distance with the 6Å to 9.64Å wavelength band offering a cumulative momentum transfer coverage of 0.006–0.19 Å^−1^. SANS data reduction was performed with the *drtsans* software^56^. Scattering intensity was corrected for detector sensitivity, background scattering and transmission values, and was consequently circularly averaged. The sample thickness was 2 mm, and the sample transmission ranged from 76.5% to 77.6% for the different wavelength. The results most relevant to the NSE experiments were a set of measurements measured on the same sample during continuous soaking in D_2_O, *i*.*e*, the data presented in Fig. 2.

### NSE experiment and data reduction

To investigate the collective dynamics we used SNS-NSE, the NSE spectrometer at the Spallation Neutron Source^57,58^, Oak Ridge National Laboratory. Measurements were carried out in 4 mm-path quartz cells at 20 °C. The NSE data acquisition setup included a combination of incident wavelengths of 8 Å and 11 Å, accessing a dynamical range of 0.1 ≤ *τ*_max_ ≤ 130ns Fourier time for different momentum transfers. Five solid angles determining *q*’s between 0.035 Å^−1^ and 0.12 Å^−1^ were set in the spin-echo measurement as *q* minimum values, and *q*’s between 0.04 Å^−1^ and 0.13 Å^−1^ were obtain from the data reduction (after *time-of-flight* grouping), that covered most of the Bragg peaks observed in the SANS experiment in Fig. 2. Graphite foil and Al_2_O_3_ were used as a standard elastic reference for data normalization and pure D_2_O was used as solvent for background subtraction. The entire experiment was ~ 130 h long, with a change of sample half-time in between, to provide fresh duckweed plants.

The NSE raw data from the two duckweed samples were combined and reduced using *DrSpine* SNS-NSE reduction software^59^. For the analysis the intermediate scattering functions were modeled using the python package PySEN^60^ developed by P. Zolnierczuk for the SNS-NSE data analysis, and *Jscatter* software^52,53^ developed by R. Biehl. The transmission ranged between 80% > transmission > 60% as a function of wavelength. Additional information about the samples scattering and transmission can be found in Supporting Information, Figure S2 and Figure S3.

## Supplementary Information

Below is the link to the electronic supplementary material.

Supplementary Material 1

## Data Availability

The datasets used and analyzed during the current study are available from the corresponding author upon reasonable request.
