# Supplementary material for: Neutrons reveal the dynamics of leaf thylakoids in living plants
Source: Sci Rep. 2025 Nov 5;15:38810. doi: 10.1038/s41598-025-22747-z (PMC12589486; doi:10.1038/s41598-025-22747-z)
Supplement: Supplementary file 1 — Supplementary Material 1 [file 41598_2025_22747_MOESM1_ESM.docx]

**SUPPORTING INFORMATION FOR MANUSCRIPT:**

**Neutrons reveal the dynamics of leaf thylakoids in living plants.**

**Authors:** Laura-Roxana Stingaciu^1*^, Hugh O’Neill^1^, Chung-Hao Liu^1^, Barbara R. Evans^2^, Gergely Nagy^1^

**Affiliations:**

^1^Neutron Scattering Division, Oak Ridge National Laboratory, Oak Ridge, TN 37831, USA.

^2^Chemical Sciences Division, Oak Ridge National Laboratory, Oak Ridge, TN 37831, USA.

*Correspondence to: [stingaciulr@ornl.gov](mailto:stingaciulr@ornl.gov)

**Figure S1. Schematic of** **duckweed leaf cell structure and chloroplast.** Starting from the left side the figure shows the duckweed plant with leaf cells; a single leaf cell enlarged to observe the cell continents; the chloroplast disk enlarged to observe the thylakoidal architecture; and an example of thylakoid membranes stack. *Note: dimensions are not too scale*.

In duckweed leaf cells photosynthesis takes place in the chloroplasts. Chloroplasts contain thylakoid membranes arranged in stacks of disks called grana (singular: granum). Grana are connected by stromal thylakoids called lamellae, which join multiple granum stacks together as a labyrinth of chambers forming a single functional compartment. The space between the thylakoids and the chloroplast membranes is called the stroma. The stroma fluid contains other components like chloroplast DNA, ribosomes, various enzymes and starch. Thylakoid membrane surrounds the thylakoid lumen separating it from the rest of the stroma fluid, therefore, forming a barrier between two different aqueous phases. The space between two adjacent thylakoid membranes is called interthylakoidal space. In leaf’s the interthylakoidal space is part of the stroma, the fluid where the light-independent reaction process of photosynthesis takes place like the carbon cycle. The thylakoid membranes have a high density of imbedded proteins that form the backbone of photosynthesis apparatus like photosystem II complex, photosystem I complex, ATP synthase and cytochromes among others.

**
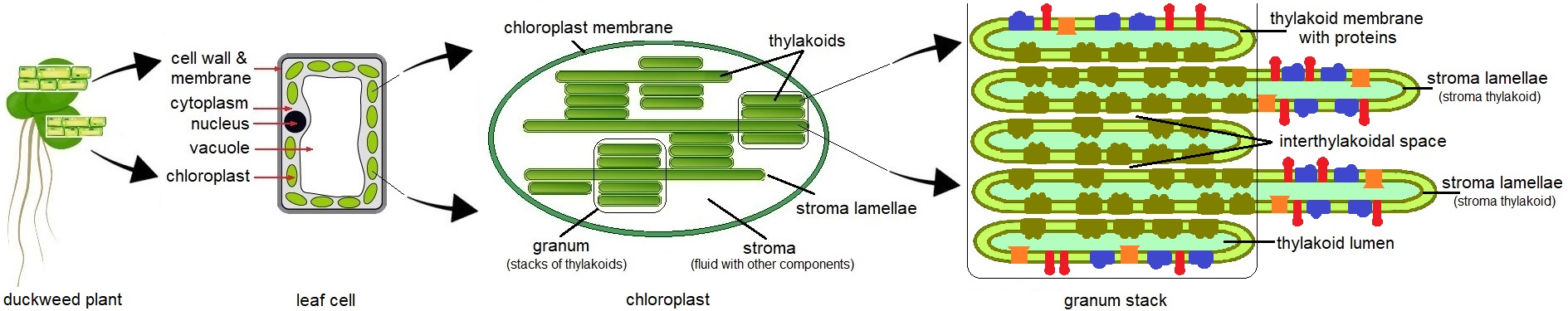
**

**Figure S2. Coherent and incoherent signal in duckweed samples measured by diffraction scans.** On the left side the figure shows the Spin Up and Spin Down signal, and on the right side the flip ratio, FR, as a function of q, for 8Å measurements.

In spin-echo spectroscopy the “Spin Up” state is mainly coherent signal, as the probability that only 1/3 of the incoherent signal will preserve spin and contribute to this state: I_up_ = I_coherent_+ 1/3I_incoherent_. “Spin Down” state is mainly incoherent: I_down_ = 2/3I_incoherent_. The ratio I_up_ / I_down_ = FR is called the Flip Ratio and is a measure of the echo intensity. From the total FR,one can see at q > 0.1Å^-1^ how the incoherent contribution to the signal becomes significant. Nonetheless, there is still sufficient coherent signal contributing, and all the echoes for these measurements are coherent (positive echoes): see FR = 1.7 - 1.25 at q = 0.1Å^-1^ - 0.14 Å^-1^. Samples have nearly identical flip ratio.

**
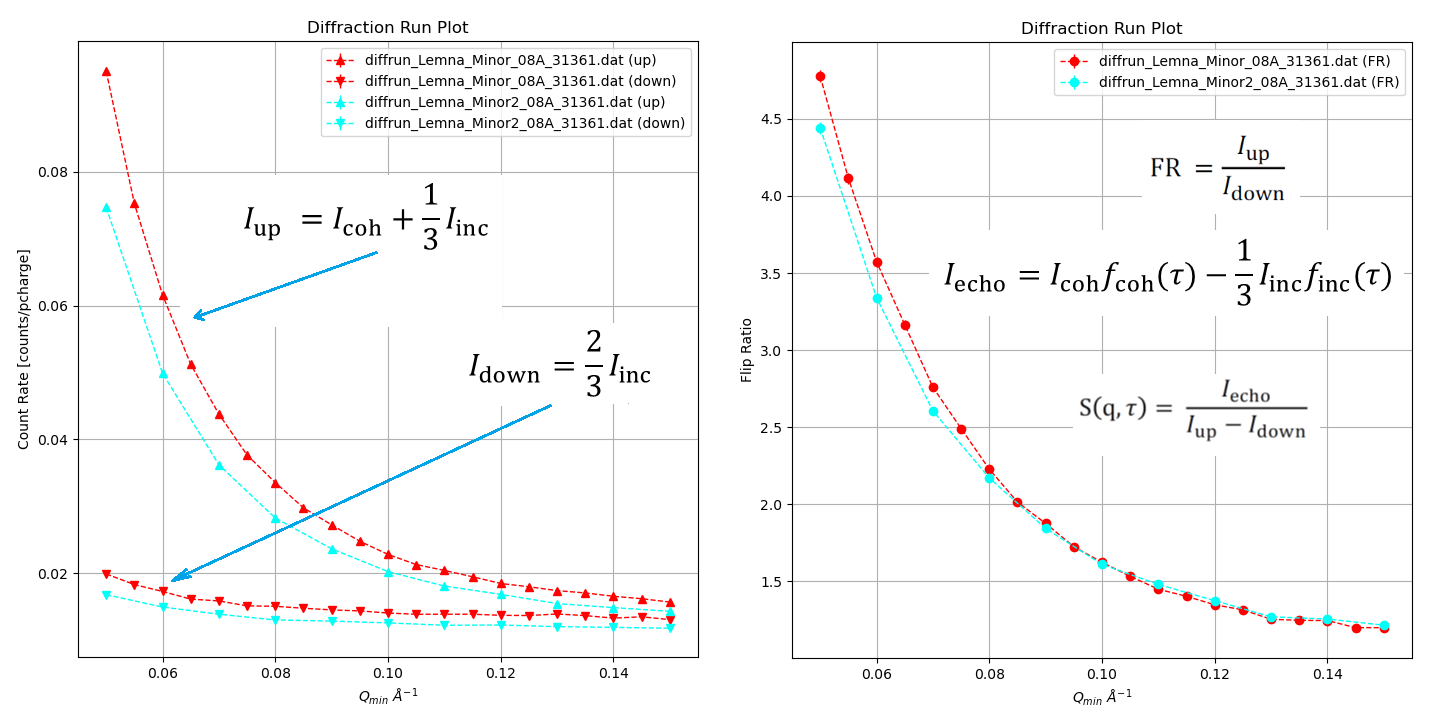
**

**Figure S3. Transmission measurements of duckweed samples and D_2_O-buffer with corresponding transmission factors.** In NSE the transmission measurements are used to weigh the sample and background contributions to the scattering signal.

Du to the nature of the neutron beam, at the SNS-NSE spectrometer the transmission is a spectrum function of the wavelenght, typically measured in 42 time-channels. During the data reduction the first and last 4 channels are excluded due to the posibility of contamination with other wavelenght band. The transmission for both solvent and samples are measured, and the ratio Tsample/Tbuffer (Tsam/T0 in the lower panel) = transmission factor (usually ≤ 1) is used in the reduction for proper weighing. The circled areas are the excluded time channels. The difference in the transmission of the two samples comes from density variations. The data for each sample was reduced by using its corresponding transmission factor to account for these differences.

**
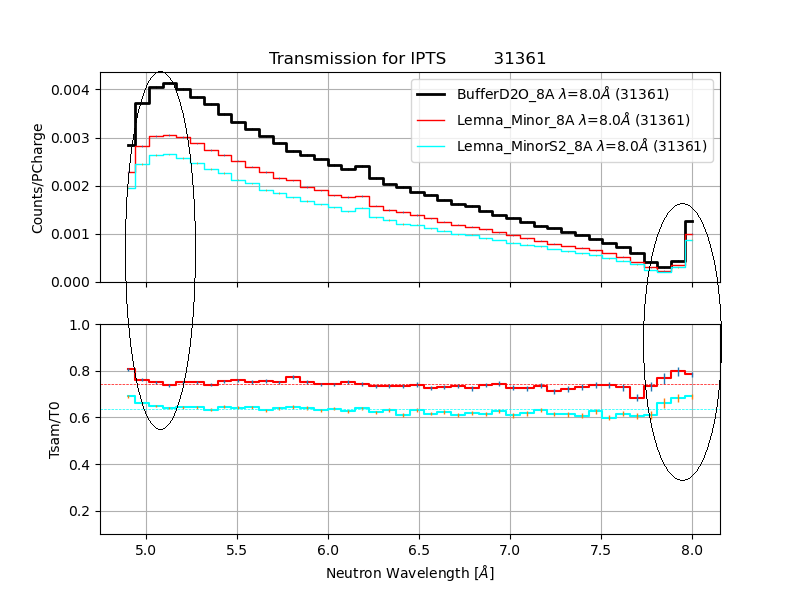
**

**Figure S4. Viability test of duckweed plants during extended soaking time in D_2_O.** The sample was prepared and loaded in the same manner as the samples used in the Neutron Spin-Echo (NSE) experiment.

**
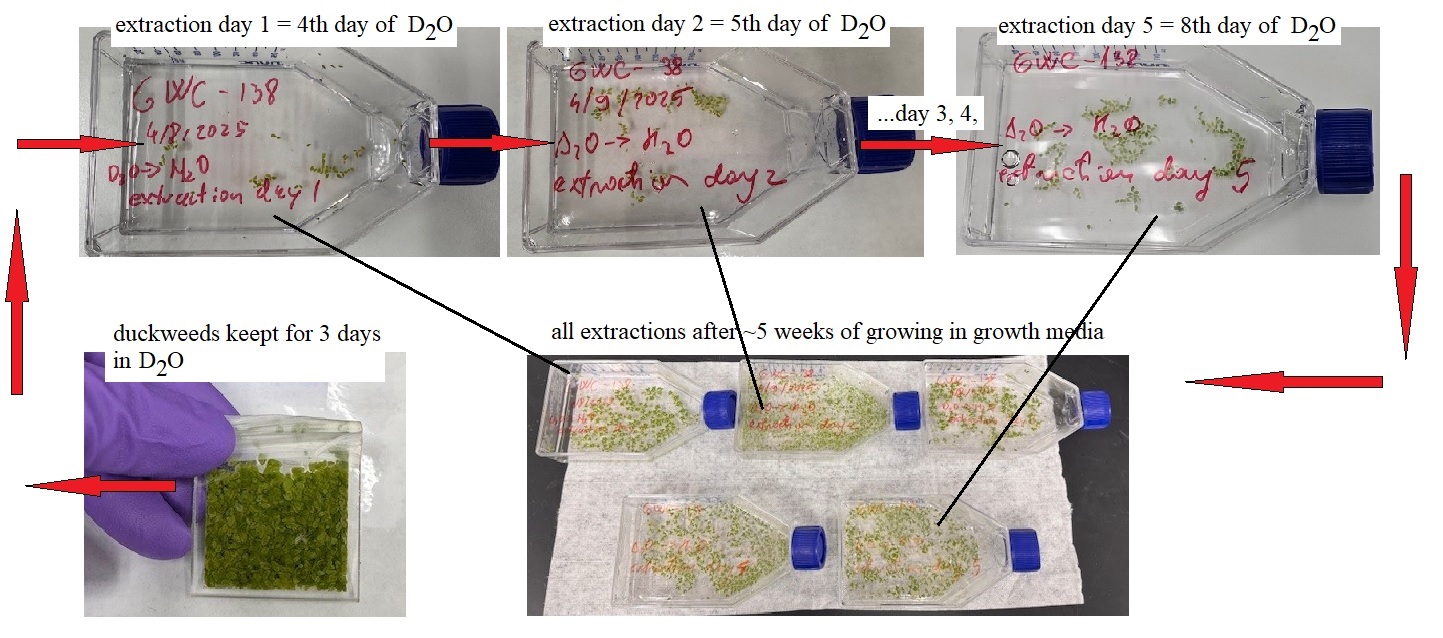
**

A duckweed sample was cultivated from the same batch of plants used in the NSE experiment. This sample was exchanged in D_2_O, loaded, and sealed in a 4 mm quartz cell in the same manner as the NSE samples (left bottom corner). It was maintained under stable conditions simulating the NSE experiment on the bench-top for three days (25°C in pure D_2_O)^*^. On the fourth day of D_2_O immersion, a small batch of plants was extracted from the NSE cell and placed in a growth bottle filled with water-based growing media. This extraction procedure was repeated daily for five consecutive days named extraction day 1 – 5 in the pictures. Notably, the plants extracted on the fifth day had survived in D_2_O for over eight days. All extractions were placed in a climate-controlled grow chamber and allowed to recover for more than five weeks. By the end of the recovery period, the density of leaves in each bottle had significantly increased due to the growth of new plants. Duckweed is known to reproduce through leaf division when alive and thriving. The increase in leaf density in each extraction bottle provides experimental evidence that even after eight days of continuous immersion in D_2_O, the plants remained alive, active, and capable of photosynthesis, enabling their multiplication.

This bench-top experiment demonstrates that, by the end of the NSE experiment, there is a very high probability that the two samples measured for a total of 65 hours each, *i*.*e*., less than three days of D_2_O exposure, are still living and actively performing photosynthesis.

*Note that this control sample was not exposed to neutron beam.
